# Determining the mechanism of pulsatilla decoction for treating gastric cancer: a network pharmacology-based study

**DOI:** 10.3389/fonc.2023.1174848

**Published:** 2023-06-09

**Authors:** Siqi Huang, Manying Qu, Xiaowu Chen, Shaochen Yu, Fanhua Kong

**Affiliations:** ^1^Department of Gastroenterology, Guangdong Second Provincial General Hospital, Guangzhou, Guangdong, China; ^2^Department of Respiratory Medicine, Guangdong Second Provincial General Hospital, Guangzhou, Guangdong, China; ^3^Department of Emergency and Critical Care Medicine, Guangdong Second Provincial General Hospital, Guangzhou, Guangdong, China; ^4^Zhongnan Hospital of Wuhan University, Institute of Hepatobiliary Diseases of Wuhan University, Transplant Center of Wuhan University, National Quality Control Center for Donated Organ Procurement, Hubei Key Laboratory of Medical Technology on Transplantation, Hubei Clinical Research Center for Natural Polymer Biological Liver, Hubei Engineering Center of Natural Polymer-based Medical Materials, Wuhan, Hubei, China

**Keywords:** *Pulsatilla decoction*, network pharmacology, traditional Chinese medicine, proliferation, apoptosis, gastric cancer

## Abstract

**Background and aim:**

Gastric cancer (GC) is a prevalent malignancy worldwide. *Pulsatilla decoction* (PD), a traditional Chinese medicine formula, can treat inflammatory bowel disease and cancers. In this study, we explored the bioactive components, potential targets, and molecular mechanisms of PD in the treatment of GC.

**Methods:**

We conducted a thorough search of online databases to gather gene data, active components, and potential target genes associated with the development of GC. Subsequently, we conducted bioinformatics analysis utilizing protein–protein interaction (PPI), network construction, and Kyoto Encyclopedia of Genes and Genomes (KEGG) to identify potential anticancer components and therapeutic targets of PD. Finally, the efficacy of PD in treating GC was further validated through *in vitro* experiments.

**Results:**

Network pharmacological analysis identified 346 compounds and 180 potential target genes associated with the impact of PD on GC. The inhibitory effect of PD on GC may be mediated through modulation of key targets such as PI3K, AKT, NF-κB, FOS, NFKBIA, and others. KEGG analysis showed that PD mainly exerted its effect on GC through the PI3K–AKT, IL-17, and TNF signaling pathways. Cell viability and cell cycle experiments showed that PD could significantly inhibit proliferation and kill GC cells. Moreover, PD primarily induces apoptosis in GC cells. Western blotting analysis confirmed that the PI3K–AKT, IL-17, and TNF signaling pathways are the main mechanisms by which PD exerts its cytotoxic effects on GC cells.

**Conclusion:**

We have validated the molecular mechanism and potential therapeutic targets of PD in treating GC through network pharmacological analysis, thereby demonstrating its anticancer efficacy against GC.

## 1 Introduction

Gastric cancer (GC) is the fifth most common cancer type and the third leading cause of death worldwide (1). The major risk factors for GC include *Helicobacter pylori* infection, age, high salt intake, and decreased intake of fruits and vegetables (2). Chronic *H. pylori* infection is the main reason for GC, accounting for approximately 89% of the distal GC cases worldwide (3). At present, >1 million people worldwide are newly diagnosed with GC each year. Although the incidence and mortality rates of GC have decreased globally over the past 50 years, it remains the third leading cause of cancer-related deaths (4). The main treatment strategy for early GC is endoscopic resection and that for non-early GC is surgery (1). Furthermore, complete surgical removal of lymph node D2 remains the main treatment strategy for GC. However, adjuvant chemotherapy after surgical resection is warranted to improve patient survival time. In the past few decades, researchers have emphasized the importance of neoadjuvant chemotherapy for GC and gastroesophageal junction and sub-esophageal adenocarcinoma, with good therapeutic effects (5). For example, a meta-analysis conducted in 2010 among patients with GC revealed that postoperative adjuvant chemotherapy using fluorouracil markedly decreased the mortality rate of these patients compared with surgery alone (6). In addition, radiation and targeted therapies play essential roles in improving the survival of patients with GC (7). However, owing to the high heterogeneity of GC, the overall prognosis of patients with advanced GC remains poor (8). At present, there are limited treatment strategies for GC, and the curative effect is poor; therefore, new therapeutic methods need to be urgently developed.

As a unique biomedical resource, traditional Chinese medicine (TCM) is widely used in the prevention and treatment of various malignant tumors (9). In addition, TCM is composed of a variety of components, which has certain advantages, including the synergistic effect of various components in the treatment of tumors (10, 11). Natural compounds extracted from herbs are considered ideal candidates for the suppression of malignant tumors due to their advantages of multiple targets and low toxicity (12). Chinese herbal compounds have been shown to be effective tumor suppressors in cancer by regulating cell proliferation, cell cycle, apoptosis, autophagy, cell senescence, epithelial–mesenchymal transformation, metastasis, angiogenesis, immune function, and chemotherapy resistance (13). *Pulsatilla decoction* (PD), consisting of *Radix Pulsatillae*, *Cortex Phellodendri*, *Rhizoma Coptidis*, and *Cortex Fraxini*, is a TCM used to treat diseases such as inflammatory bowel disease and colon cancer (14, 15). PD can alleviate acute colitis in mice by inhibiting inflammation and destroying the epithelial barrier (16). Currently, there are only a few studies on the potential application of PD in the treatment of malignant tumors. PD was reported to increase the immunogenic cell death of colon cancer cells induced by 5-FU, and the combination of 5-FU and PD was found to improve the anti-tumor activity of 5-FU in colorectal cancer (14). However, the molecular mechanism of PD in the treatment of GC remains unknown.

At present, no comprehensive studies on the use of Pulsatilla decoction (PD) for treating GC are available, warranting further exploration of its therapeutic effects and mechanisms. Network pharmacology, a hot topic in pharmacological research, is a new drug research method based on the multidirectional analysis of systems pharmacology and biology (11). The network pharmacology of TCM is suitable for characterizing its multitarget and complex pharmacological actions, thereby providing a good prediction platform for determining the mechanisms of TCMs (17). Furthermore, it can target multiple nodes in the interacting molecular systems, thereby improving efficacy and reducing adverse reactions (17, 18).

**Figure 1:**
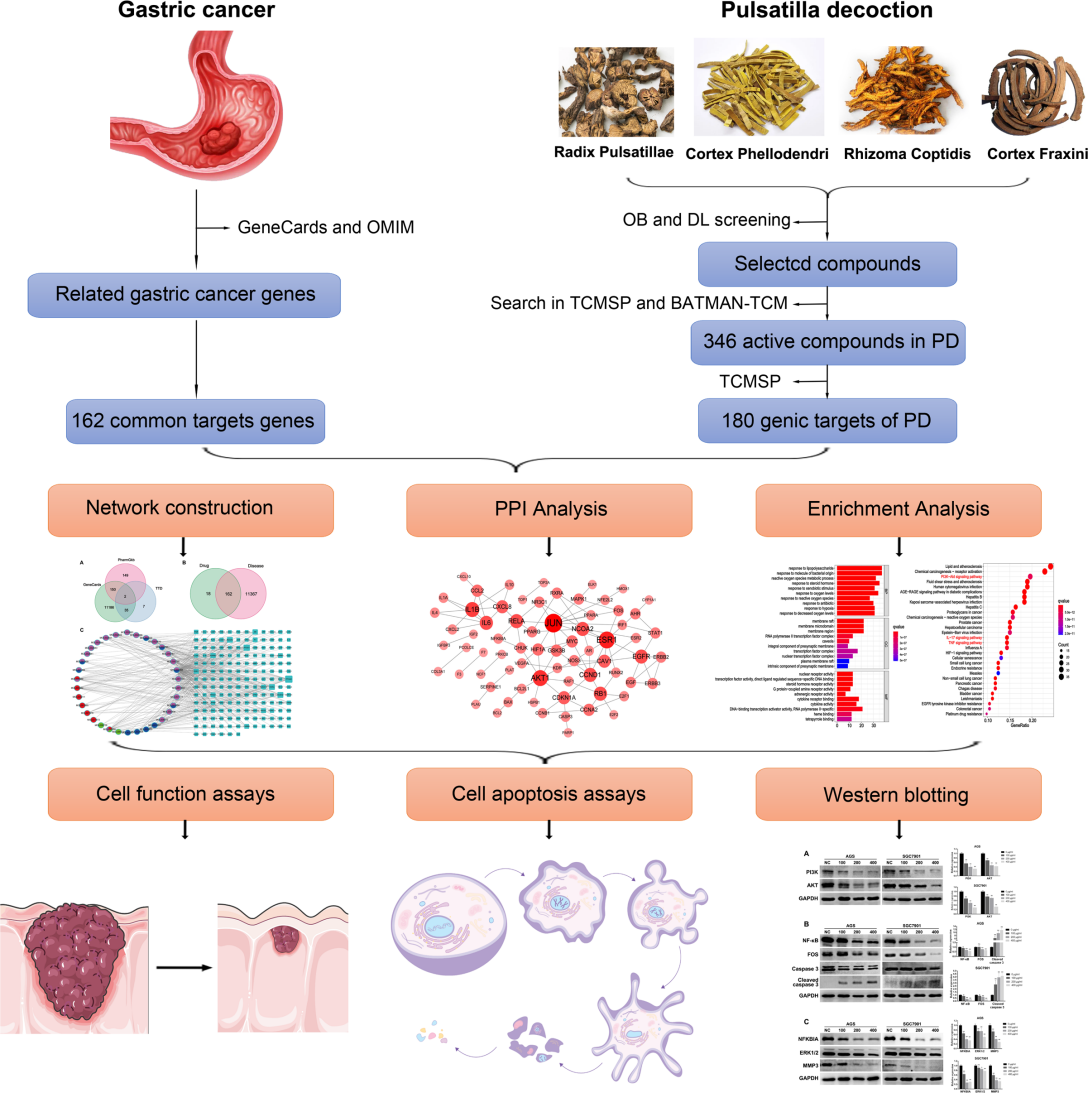
The technical strategy used in this study. The active components and potential target genes of PD were analyzed by network pharmacology. Network structure, PPI, and enrichment analysis were further analyzed. Finally, *in vitro* experiments were conducted to verify the therapeutic effect of PD on GC and explore its potential molecular mechanism.

In the present study, the active components and targets in PD were studied for the first time. Using network pharmacology, we identified the potential therapeutic targets of PD and verified the results *via* controlled trials. This study aims to lay a foundation for further elucidating the mechanism of action of PD for treating GC and to provide a basis for its extensive clinical applications. **Figure 1** presents the detailed technical strategies used in the present study.

## 2 Materials and methods

### 2.1 Data preparation

#### 2.1.1 Composition of PD

Two public databases, namely, Traditional Chinese Medicines for Systems Pharmacology Database and Analysis Platform (TCMSP, available online: https://old.tcmsp-e.com/tcmsp.php) and Bioinformatics Analysis Tool for Molecular mechANism of Traditional Chinese Medicine (BATMAM-TCM, available online: http://bionet.ncpsb.org.cn/batman-tcm/), were used to retrieve the ingredients in the four herbs of PD.

#### 2.1.2 Ingredient targets in PD and GC-associated targets

TCMSP was used to identify the ingredient targets in the four herbs of PD; these targets were imported into the UniProt database to convert the protein target names to their official gene symbols. The GC-associated targets were obtained from the GeneCards (https://www.genecards.org/, ver, 5.11), Therapeutic Target Database (TTD, http://db.idrblab.net/ttd/, updated on 29 September 2021), and PharmGkb (https://www.pharmgkb.org/) databases. Thereafter, the intersection genes of PD and GC were obtained, which were the target genes for PD treating GC.

### 2.2 Bioinformatics analysis

#### 2.2.1 Protein–protein interaction data

Protein–protein interaction (PPI) analysis was conducted by submitting the intersection genes to the online Search Tool for the Retrieval of Interacting Genes/Proteins (STRING) database (https://cn.string-db.org/); the species was chosen as *Homo sapiens*. The minimum required interaction score was >0.990, and disconnected nodes were hidden in the network. Then, the TSV file, containing the results of the PPI analysis, was downloaded from STRING and imported into Cytoscape 3.7.1, which is widely used to construct and visualize networks. Degree, a key topological parameter, was used to characterize the most significant nodes in the network and was calculated using the CytoNCA plugin in the Cytoscape software. The more significant the node, the higher the quantitative values of the topological parameters.

#### 2.2.2 Enrichment analysis

Gene Ontology (GO) and Kyoto Encyclopedia of Genes and Genomes (KEGG) pathway enrichment analyses were conducted using tools available in the R package, such as clusterProfiler, ggplot2, and enrichplot. A *p*-value of <0.05 was considered statistically significant and chosen for further analysis.

#### 2.2.3 Network construction

Three networks were constructed and visualized using Cytoscape 3.7.1. The networks were as follows: (1) bioactive compound–disease–target network (C–D–T) of PD treatment for GC; (2) PPI network of PD treatment for GC; and (3) bioactive compound–pathway–target (C–P–T) network.

#### 2.2.4 Molecular docking

Molecular docking is a common technology for network pharmacology owing to its characteristic of precisely predicting the conformation of small-molecule ligands within the target binding site and assessing the binding affinity. The candidate target proteins and compounds present in *H. sapiens* and with higher degree values in the PPI and C–D–T networks were selected for molecular docking. The Molecular Operating Environment (MOE) (v2015.10) software was used for molecular docking to validate compound–target interaction. Molecular docking was performed as follows. First, the three-dimensional (3D) structure of the candidate proteins was obtained from Protein Data Bank (PDB) (http://www.rcsb.org/), with the species limited to *Homo sapiens*, and the structure of the compound was downloaded from PubChem (https://pubchem.ncbi.nlm.nih.gov/). Second, the 3D structure of the candidate protein was imported into MOE to construct the mating pockets after removing water molecules, preparing the protein structure, and minimizing energy. Lastly, mating pocket was performed molecular docking with the candidate compound.

### 2.3 Experimental validation

#### 2.3.1 Preparation of the PD extract

*Radix Pulsatillae* (20 g), *Cortex Phellodendri* (16 g), *Rhizoma Coptidis* (8 g), and *Cortex Fraxini* (16 g) were purchased from the Pharmacy of Traditional Chinese Medicine of the Second Xiangya Hospital, Central South University. The PD water extract was prepared by immersing the abovementioned herbs in eight volumes of distilled water for 30 min, followed by decoction for 1.5 h, centrifugation at 10,000 *g* for 30 min, and collection of the supernatants. The extraction step was repeated two times. The obtained supernatants were mixed to evaporate the solvent and vacuum dried to a powder. Then, the powder was dissolved in dimethyl sulfoxide (200 mg/ml). The solution was filtered through a 0.22-µm filter to remove insoluble matter. Finally, the solution was divided and stored at −20° until further use.

#### 2.3.2 Cell lines and culture

The AGS and SGC7901 cell lines were purchased from the Cell Bank of the Chinese Academy of Sciences (Shanghai, China). All cells were maintained in RPMI 1640 (Gibco, El Paso, TX, USA) supplemented with 10% fetal bovine serum (Gibco). The cells were cultured in a humidified incubator at 37°C with 5% CO_2_.

#### 2.3.3 Cell viability assay

AGS and SGC7901 cells were inoculated into 96-well plates at a density of 1 × 10^4^ cells/well. After culturing the cells at 37°C for 24 h, they were treated with different concentrations of PD (0, 100, 200, 300, 400, 500, 600, 700, 800, 900, and 1,000 μg/ml). Cell viability was measured using the Cell Counting Kit-8 (CCK-8) reagent (Genview, USA) after 24, 48, and 72 h. All data were normalized to those of the control without cells and expressed as mean ± SD.

#### 2.3.4 Colony formation assay

AGS and SGC7901 cells were inoculated into six-well plates at a density of 5 × 10^3^ cells/well. After culturing the cells at 37°C for 24 h, they were treated with different concentrations of PD (0, 200, and 400 μg/ml) for 72 h. Then, the cells were fixed with 4% paraformaldehyde for 30 min, stained with crystal violet solution for 2 h, washed with PBS, and manually photographed.

#### 2.3.5 Flow cytometric analysis of the cell cycle

AGS and SGC7901 cells were inoculated into six-well plates at a density of 3 × 10^5^ cells/well. After culturing the cells at 37°C for 24 h, they were treated with different concentrations of PD (0, 100, 200, and 400 μg/ml) for 48 h. The cells were digested with pancreatic enzymes and collected. Then, the samples were fixed with 70% ethanol at 4° overnight and stained with propyl iodide (PI, 50 μg/ml; Genview) in the dark for 45 min. Cell cycle analysis was performed using the Canto II flow cytometer (BD Bioscience, USA).

#### 2.3.6 Flow cytometric analysis of cell apoptosis

AGS and SGC7901 cells were inoculated into six-well plates at a density of 3 × 10^5^ cells/well. After culturing the cells at 37°C for 24 h, they were treated with different concentrations of PD (0, 200, 400, and 600 μg/ml) for 48 h. Then, the cells were digested with EDTA-free pancreatic enzymes and collected. Subsequently, 5 µl of annexin V–fluorescein isothiocyanate (FITC) and 5 µl of the PI staining solution (Genview) were added and the samples were incubated for 15 min at 15-30°C in the dark. Apoptosis was detected using a flow cytometer (BD Bioscience). The data were analyzed using FlowJo 7.6 software (*De Novo* Software, Los Angeles, CA, USA).

#### 2.3.7 Western blotting

AGS and SGC7901 cells were inoculated into six-well plates at a density of 3 × 10^5^ cells/well. After culturing the cells at 37°C for 24 h, they were treated with different concentrations of PD (0, 100, 200, and 400 μg/ml) for 48 h. The cells were collected using cell scrapers, and total proteins were extracted from the cell lysate. The denatured proteins were added to the electrophoresis chamber for an appropriate amount of time and then transferred onto a polyvinylidene difluoride membrane. The membrane was blocked with 5% bovine serum albumin for 2 h at room temperature. Then, the membrane was incubated with PI3K (1:1,000, ABclonal, Cat No: A0265), AKT (1:1,000, ABclonal, Cat No: A17909), NF-κB (1:1,000, ABclonal, Cat No: A6667), FOS (1:1,000, Proteintech, Cat No: 66590-1-Ig), caspase 3 (1:1,000, Proteintech, Cat No: 19677-1-AP), NFKBIA (1:1,000, ABclonal, Cat No: A11397), ERK1/2 (1:1,000, Proteintech, Cat No: 16443-1-AP), MMP3 (1:1,000, Proteintech, Cat No: 17873-1-AP), and GAPDH (1:10,000, Proteintech, Cat No: 60004-1-Ig) overnight at 4°C. Thereafter, the membrane was incubated with ABclonal antibody (1:5,000) for 1 h. The fluorescence intensity of the bands was detected using an enhanced chemiluminescence detection kit (Genview). The bands were quantitatively analyzed using ImageJ software (Version 11).

### 2.4 Ethics approval

This study was approved by the Medical Ethics Committee of Guangdong Second Provincial General Hospital. All experiments were conducted following the study protocol.

### 2.5 Statistical analysis

Statistical analysis was performed using the GraphPad Prism 6 software (La Jolla, CA, USA). The data are expressed as the mean ± standard deviation and were analyzed using Student’s *t*-test. Differences between groups were considered statistically significant at *p* < 0.05.

## 3 Results

### 3.1 Compounds and screening of PD

In total, 346 PD compounds were collected from the TCMSP and BATMAM-TCM databases, of which 71 were from *Rhizoma Coptidis*, 27 were from *Cortex Fraxini*, 177 were from *Cortex Phellodendri*, and 71 were from *Radix Pulsatillae*. All PD compounds and their corresponding parameters, such as absorption, distribution, metabolism, and excretion, were obtained from the TCMSP database. The criteria to screen the compounds for subsequent analysis were as follows: oral bioavailability of ≥30% and DL value of ≥0.18 (**Table 1**).

**Table 1:** Compounds of pulsatilla decoction.

MOL ID	MOL Name	OB	DL	Medicine	
**MOL006710**	8-(beta-D-Glucopyranosyloxy)-7-hydroxy-6-methoxy-2H-1-benzopyran-2-one	36.76	0.42	Cortex Fraxini	TCMSP
**MOL006709**	AIDS214634	92.43	0.55	Cortex Fraxini	TCMSP
**MOL000358**	beta-Sitosterol	36.91	0.75	Cortex Fraxini, Radix Pulsatillae, Cortex Phellodendri	TCMSP
**MOL001455**	(S)-Canadine	53.83	0.77	Cortex Phellodendri	TCMSP
**MOL005438**	Campesterol	37.58	0.71	Cortex Phellodendri	TCMSP, BATMAM-TCM
**MOL002671**	Candletoxin A	31.81	0.69	Cortex Phellodendri	TCMSP, BATMAM-TCM
**MOL002670**	Cavidine	35.64	0.81	Cortex Phellodendri	TCMSP
**MOL002666**	Chelerythrine	34.18	0.78	Cortex Phellodendri	TCMSP
**MOL002651**	Dehydrotanshinone II A	43.76	0.4	Cortex Phellodendri	TCMSP
**MOL002643**	delta 7-Stigmastenol	37.42	0.75	Cortex Phellodendri	TCMSP
**MOL002652**	delta 7-Dehydrosophoramine	54.45	0.25	Cortex Phellodendri	TCMSP, BATMAM-TCM
**MOL002656**	Dihydroniloticin	36.43	0.81	Cortex Phellodendri	TCMSP
**MOL006392**	Dihydroniloticin	36.43	0.82	Cortex Phellodendri	TCMSP
**MOL000787**	Fumarine	59.26	0.83	Cortex Phellodendri	TCMSP
**MOL002672**	Hericenone H	39	0.63	Cortex Phellodendri	TCMSP, BATMAM-TCM
**MOL002673**	Hispidone	36.18	0.83	Cortex Phellodendri	TCMSP
**MOL000790**	Isocorypalmine	35.77	0.59	Cortex Phellodendri	TCMSP
**MOL002636**	Kihadalactone A	34.21	0.82	Cortex Phellodendri	TCMSP
**MOL002659**	Kihadanin A	31.6	0.7	Cortex Phellodendri	TCMSP
**MOL006401**	Melianone	40.53	0.78	Cortex Phellodendri	TCMSP
**MOL002660**	Niloticin	41.41	0.82	Cortex Phellodendri	TCMSP, BATMAM-TCM
**MOL001131**	Phellamurin_qt	56.6	0.39	Cortex Phellodendri	TCMSP
**MOL002641**	Phellavin_qt	35.86	0.44	Cortex Phellodendri	TCMSP
**MOL006413**	Phellochin	35.41	0.82	Cortex Phellodendri	TCMSP
**MOL002644**	Phellopterin	40.19	0.28	Cortex Phellodendri	TCMSP, BATMAM-TCM
**MOL001771**	Poriferast-5-en-3beta-ol	36.91	0.75	Cortex Phellodendri	TCMSP
**MOL002662**	Rutaecarpine	40.3	0.6	Cortex Phellodendri	TCMSP
**MOL002663**	Skimmianin	40.14	0.2	Cortex Phellodendri	TCMSP
**MOL006422**	Thalifendine	44.41	0.73	Cortex Phellodendri	TCMSP, BATMAM-TCM
**MOL001454**	Berberine	36.86	0.78	Cortex Phellodendri, Rhizoma Coptidis	TCMSP, BATMAM-TCM
**MOL002894**	Berberrubine	35.74	0.73	Cortex Phellodendri, Rhizoma Coptidis	TCMSP
**MOL001458**	Coptisine	30.67	0.86	Cortex Phellodendri, Rhizoma Coptidis	TCMSP, BATMAM-TCM
**MOL000622**	Magnograndiolide	63.71	0.19	Cortex Phellodendri, Rhizoma Coptidis	TCMSP, BATMAM-TCM
**MOL013352**	Obacunone	43.29	0.77	Cortex Phellodendri, Rhizoma Coptidis	TCMSP
**MOL000785**	Palmatine	64.6	0.65	Cortex Phellodendri, Rhizoma Coptidis	TCMSP, BATMAM-TCM
**MOL000762**	Palmidin A	35.36	0.65	Cortex Phellodendri, Rhizoma Coptidis	TCMSP, BATMAM-TCM
**MOL000098**	Quercetin	46.43	0.28	Cortex Phellodendri, Rhizoma Coptidis	TCMSP
**MOL002668**	Worenine	45.83	0.87	Cortex Phellodendri, Rhizoma Coptidis	TCMSP, BATMAM-TCM
**MOL001984**	3beta,23-Dihydroxy-lup-20 (29)-ene-28-O-alpha-L-rhamnopyranosyl- (1–4)-beta-D-glucopyranosyl (1–6)-beta-D-glucopyranoside_qt	37.59	0.79	Radix Pulsatillae	TCMSP
**MOL001978**	Aureusidin	53.42	0.24	Radix Pulsatillae	TCMSP
**MOL000354**	Isorhamnetin	49.6	0.31	Radix Pulsatillae	TCMSP
**MOL001979**	LAN	42.12	0.75	Radix Pulsatillae	TCMSP
**MOL000211**	Mairin	55.38	0.78	Radix Pulsatillae	TCMSP
**MOL001971**	Pulchinenoside C_qt	37.79	0.76	Radix Pulsatillae	TCMSP
**MOL001973**	Sitosteryl acetate	40.39	0.85	Radix Pulsatillae	TCMSP
**MOL001985**	ZINC01615307	56.38	0.87	Radix Pulsatillae	TCMSP
**MOL001987**	β-Sitosterol	33.94	0.7	Radix Pulsatillae	TCMSP
**MOL000449**	Stigmasterol	43.83	0.76	Radix Pulsatillae, Cortex Phellodendri	TCMSP
**MOL002903**	(R)-Canadine	55.37	0.77	Rhizoma Coptidis	TCMSP
**MOL002904**	Berlambine	36.68	0.82	Rhizoma Coptidis	TCMSP
**MOL002907**	Corchoroside A_qt	104.95	0.78	Rhizoma Coptidis	TCMSP
**MOL002897**	Epiberberine	43.09	0.78	Rhizoma Coptidis	TCMSP, BATMAM-TCM
**MOL008647**	Moupinamide	86.71	0.26	Rhizoma Coptidis	TCMSP

### 3.2 Putative target genes and C–D–T network analysis

**Figure 2:**
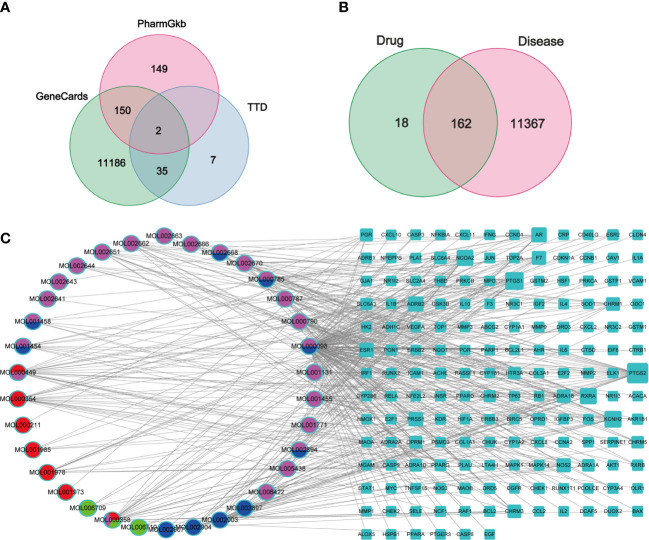
Venn diagrams and C-D-T network analysis of PD. **(A, B)** Venn diagrams of GC target genes collected from three databases **(A)** and the intersection genes of PD and GC **(B)**, respectively. **(C)** The C-D-T network of PD anti-GC. The blue rectangle represents 162 intersection genes; pink, red, blue, and green ellipse stand for compounds in *Cortex Phellodendri*, *Radix Pulsatillae*, *Rhizomah Coptidis*, and *Cortex Fraxini*, respectively.

In total, 180 putative genes of PD were collected from the TCMSP database. Furthermore, 11,373 GC-related genes were collected from GeneCards, with a relevance score of ≥1, 43 related genes were collected from TTD, and 301 related genes were collected from PharmGkb (**Figure 2A**). Finally, 162 intersection genes were retrieved as hub genes for subsequent analysis (**Figure 2B**).

The C–D–T network, comprising 196 nodes and 429 edges, was constructed to verify the relationship between PD compounds and PD and GC intersection genes, indicating a PD anti-GC effect *via* multi-compounds and multi-target interaction (**Figure 2C**). The pink ellipse represents the compounds in *Cortex Phellodendri*, the red ellipse represents the compounds in *Radix Pulsatillae*, the blue ellipse represents the compounds in *Rhizoma Coptidis*, and the green ellipse represents the compounds in *Cortex Fraxini*. The multicolor ellipse represents the common compounds in two or three herbs. The top three compounds with high degree values were quercetin (MOL000098 128), isorhamnetin (MOL000449, 22), and beta-sitosterol (MOL000358, 21); these compounds may play crucial roles in treating GC. The blue rectangle represents 130 genes; the larger the size, the higher the degree.

### 3.3 PPI network analysis

**Figure 3:**
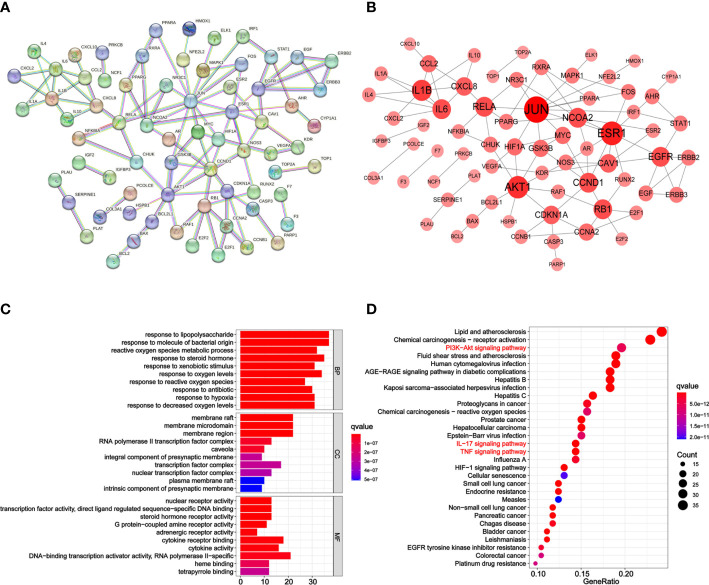
PPI network, GO, and KEGG analysis of PD anti-GC. **(A)** The PPI network acquired from the STRING database. **(B)** PPI network imported from the STRING database to Cytoscape 3.7.1. Larger and redder node sizes indicated higher degree values. **(C)** Bar plot diagram of top 10 GO terms of BP, CC, and MF. **(D)** Bubble plot diagram of KEGG enrichment pathways (top 30). The *x*-axis represents the number of target genes in each pathway and the ordinate represents each entry. Redder means lower *q*-value.

The PPI network was constructed to identify the interactions between GC and PD. The 162 intersection genes were imported into the STRING database. Then, the PPI network with hidden disconnected nodes was downloaded (**Figure 3A**). The PPI network was visualized using Cytoscape 3.7.1; it contains 70 nodes and 101 edges. A node with a larger size and a deeper red color represents a higher degree value. The top three genes with higher degree values were *JUN* (12), *ESR1* (9), and *AKT1* (8); these genes may play crucial roles in PD effects against GC (**Figure 3B**).

### 3.4 GO enrichment analysis

GO and KEGG pathway enrichment analyses were conducted to determine the functions and pathways of the 162 intersection genes using the R package. Biological process (BP), cell composition (CC), and molecular function (MF) were analyzed. In total, 2,563 GO terms were enriched from the 162 intersection genes between GC and PD. Among them, 2,302 were BP, 82 were CC, and 179 were MF terms (**Supplementary Table 1**). The top 10 significant BP, CC, and MF terms with lower adjusted *q*-values were visualized in a bar plot (**Figure 3C**). The *x*-axis represents the counts of the genes for GO enrichment terms and the *y*-axis indicates the GO terms. The red color of the bar plot represents the lower adjusted *q*-values and greater enrichment of the GO term. The results revealed that the targets of PD anti-GC, which exhibit nuclear receptor activity (GO:0004879), transcription factor activity, direct ligand-regulated sequence-specific DNA binding (GO:0098531), steroid hormone receptor activity (GO:0003707), protein-coupled amine receptor activity (GO:0008227), and adrenergic receptor activity (GO:0004935), were mainly enriched in membrane raft (GO:0045121), membrane microdomain (GO:0098857), membrane region (GO:0098589), RNA polymerase II transcription factor complex (GO:0090575), and caveola (GO:0005901) and involved in response to lipopolysaccharide (GO:0032496), response to molecule of bacterial origin (GO:0002237), reactive oxygen species metabolic process (GO:0072593), response to steroid hormone (GO:0048545), and response to xenobiotic stimulus (GO:0009410) against GC.

### 3.5 KEGG pathway analysis and C–P–T network construction

**Figure 4:**
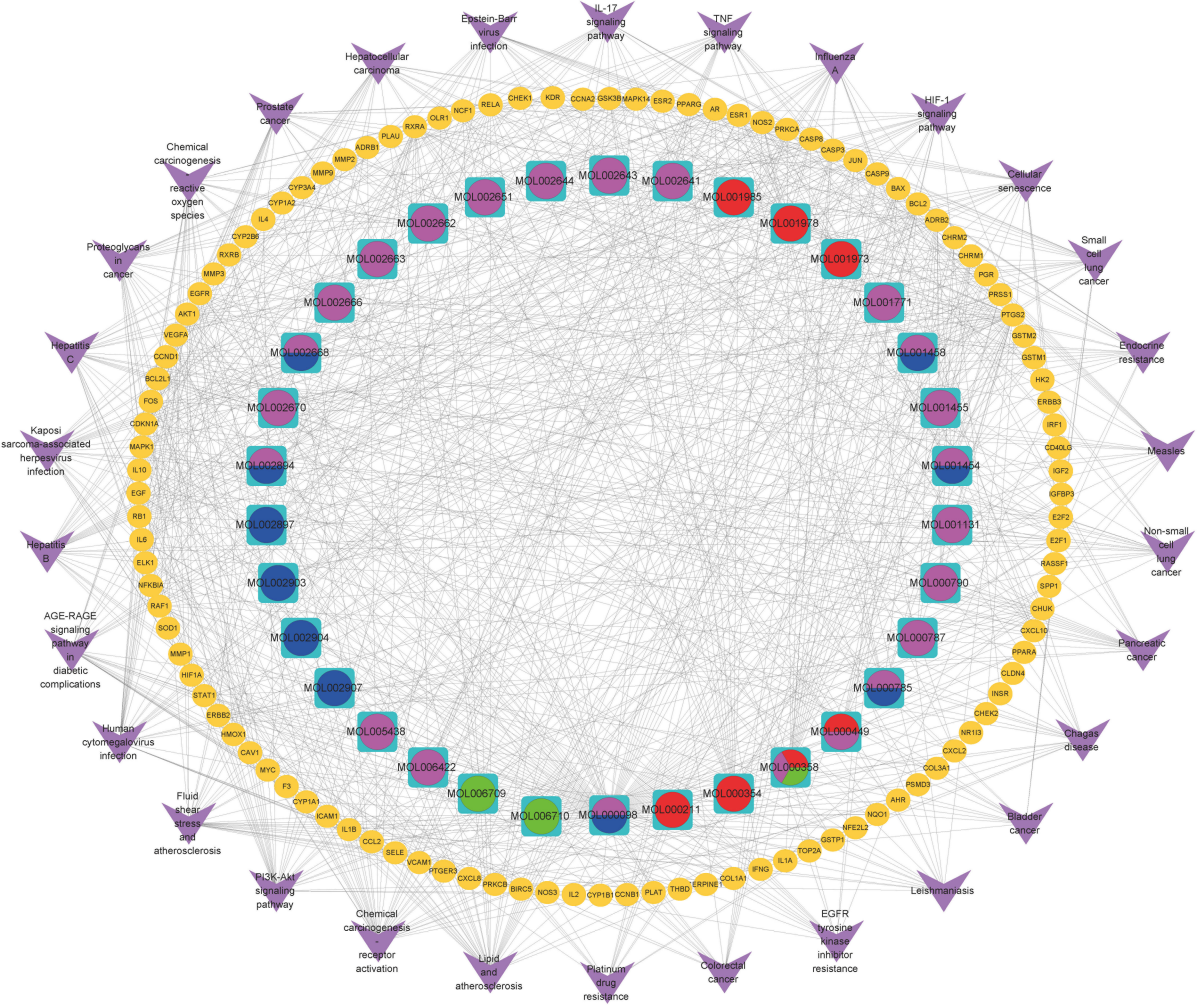
The C-P-T network of the top 30 pathways. The yellow ellipse nodes stand for genes; purple V represent pathway terms; pink, red, blue, and green ellipse stand for compounds in *Cortex Phellodendri*, *Radix Pulsatillae*, *Rhizomah Coptidis*, and *Cortex Fraxini*, respectively.

KEGG pathway enrichment analysis was performed to elucidate the pathways of the identified target genes of PD anti-GC. KEGG pathway analysis revealed that 153 of the 162 target genes were enriched, yielding a total of 176 pathways (*p* < 0.05, **Supplementary Table 2**); this suggests a PD anti-GC effect *via* multi-target genes and multi-pathways. The top 30 KEGG pathways with larger gene counts were visualized using a bubble plot (**Figure 3D**). The top three KEGG pathways with larger gene counts were lipid and atherosclerosis (hsa05417, *n* = 37), chemical carcinogenesis-receptor activation (hsa05207, *n* = 35), and the PI3K–Akt signaling pathway (hsa04151, *n* = 30). Moreover, among the top three pathways mentioned above, the PI3K–AKT (**Supplementary Figure 1**), TNF (**Supplementary Figure 2**), and IL-17 (**Supplementary Figure 3**) signaling pathways were the most associated with cancer and may be the key pathways acting on GC. Therefore, these pathways were selected for subsequent validation experiments. Interestingly, we found that the genes of PD anti-GC in the PI3K–AKT, TNF, and IL-17 signaling pathways, with red nodes representing anti-GC genes, were involved in suppressing cell proliferation, angiogenesis, DNA repair, and the cell cycle and promoting apoptosis (**Supplementary Figures 1**–**3**). To further determine the interrelationships among the compounds, pathways, and related target genes, the C–P–T network with 172 nodes and 914 edges was constructed (**Figure 4**). The results indicate a PD anti-GC effect *via* multiple pathways and target genes.

### 3.6 Molecular docking analysis

**Figure 5:**
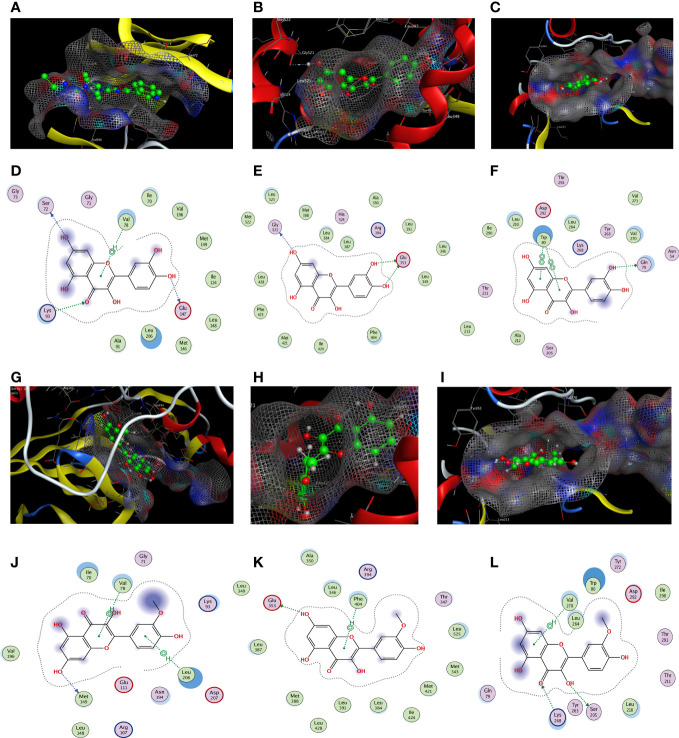
The docking model of quercetin and isorhamnetin with JUN, ESR1, and AKT1, respectively. Binding model of quercetin on the molecular surface of JUN **(A)**, ESR1 **(B)**, AKT1 **(C)**. The interaction model of quercetin with JUN **(D)**, ESR1 **(E)**, and AKT1 **(F)**. The action model of isorhamnetin with JUN **(G)**, ESR1 **(H)**, and AKT1 **(I)**. The interaction model of isorhamnetin with JUN **(J)**, ESR1 **(K)**, and AKT1 **(L)** The ligands in the binding model and interaction model are colored green.

Based on the findings of PPI and C–D–T network analyses, JUN (PDB code: 2P33), ESR1 (PDB code: 1A52), and AKT1 (PDB code: 3O96) were selected for docking experiments with quercetin and isorhamnetin owing to their higher degree values. The docking results of quercetin with JUN, ESR1, and AKT1 are demonstrated in **Figures 5A–C**. In JUN, quercetin formed three hydrogen bonds and one H–π bond with Ser72, Gly147, Lys93, and Val78, respectively (**Figure 5D**). In ESR1, quercetin formed three hydrogen bonds with Glu353 and GLy251 (**Figure 5E**). In AKT1, quercetin formed two π–π bonds with Trp80 and one hydrogen bond with Gln79 (**Figure 5F**).

The docking results of isorhamnetin with JUN, ESR1, and AKT1 are demonstrated in **Figures 5G–I**. Isorhamnetin formed two hydrogen bonds and one π–π bond with Ile290, Gln79, and Trp80, respectively, in JUN (**Figure 5J**). In ESR1, isorhamnetin formed one hydrogen bond and two π–H bonds with Met149, Val78, and Leu206, respectively (**Figure 5K**). In AKT1, isorhamnetin formed one hydrogen bond and one H–π bond with Glu353 and Phe404, respectively (**Figure 5L**).

In summary, molecular docking revealed that the ingredients and protein targets interacted *via* different bonds. The matching degree between the ingredients and proteins was assessed using binding energy (**Table 2**). The lower the binding energy, the greater the stability. These results suggest that compounds can bind well with the active sites in the protein targets.

**Table 2:** Binding energy results of molecular docking (kcal/mol).

Ligands Receptors	JUN	ESR1	AKT1
**Quercetin**	−7.4788	−6.9107	−6.8825
**Isorhamnetin**	−6.015	−5.8736	−6.6194

### 3.7 PD inhibits GC cell proliferation

**Figure 6:**
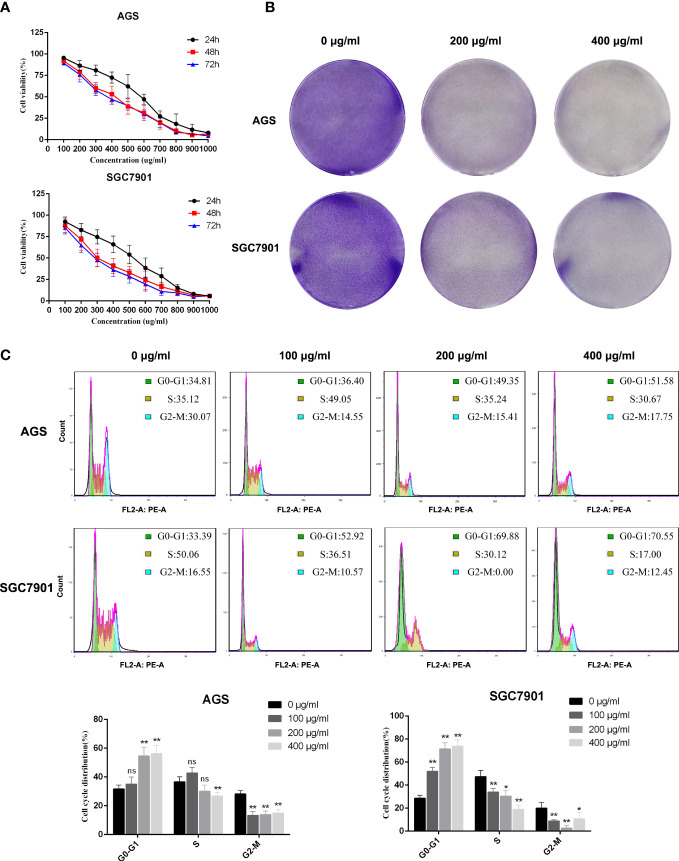
PD inhibits the proliferation of GC cells. **(A)** Cell viability assay showed that PD could decrease the viability of GC cells. The GC cells were treated with PD at different concentrations for 24, 48 and 72 h. **(B)** Representative images showing colonies formed by GC cells treated with various concentrations of PD for 3 days. **(C)** Representative images and statistical graphs of GC cell cycle analysis. *p < 0.05, **p < 0.01 versus the untreated group, ns, no significance.

To verify the results of the network pharmacological analysis of PD, AGS and SGC7901 cells were used to verify the killing effect of PD on GC. First, we verified the effects of different PD concentrations on GC *via the* CCK-8 assay. **Figure 6A** demonstrates that the survival rate of GC cells decreased as the PD dose and exposure time increased. The cell viability data suggest that PD has an obvious killing effect on GC cells; the effects of an incubation time of 48 and 72 h were better than those of 24 h; however, the difference of the effects at 48 and 72 h was not significant. In addition, we evaluated the effects of PD on the proliferation ability of GC cells using the clonal formation assay. A concentration gradient of 0, 200, and 400 μg/ml was used. The colony formation ability of AGS and SGC7901 cells was significantly reduced, and PD significantly inhibited GC cell proliferation (**Figure 6B**). Flow cytometry further confirmed delayed G1/S transition and cell cycle arrest after treating AGS and SGC7901 cells with PD (**Figure 6C**). Taken together, these results indicate that PD can significantly inhibit GC cell proliferation.

### 3.8 PD promotes GC cell apoptosis

**Figure 7:**
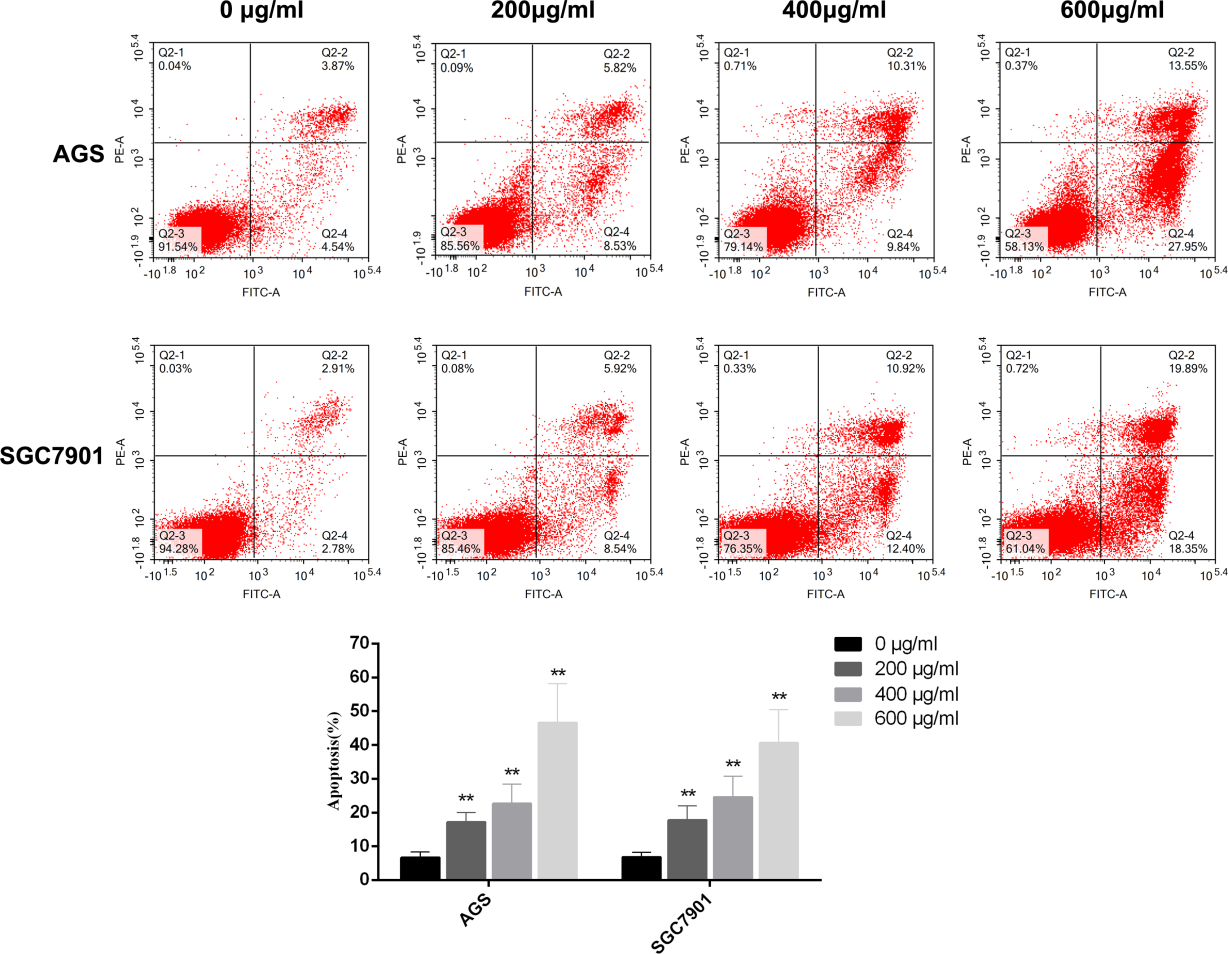
GC cells were treated with different concentrations of PD for 48 h, and the apoptosis of GC cells was detected by flow cytometry. The percentage of apoptotic cells was expressed as the mean ± SD of three independent experiments. **p < 0.01 versus the untreated group.

The cell viability assay revealed that PD has a significant killing effect on GC cells. Therefore, we performed flow cytometry to determine the effects of PD on GC cell apoptosis. We inoculated GC cells into six-well plates and incubated them with PD at different concentrations (0, 200, 400, and 600 μg/ml) for 48 h. Annexin V–FITC and PI staining was performed to detect the degree of GC cell apoptosis. As shown in **Figure 7**, the apoptosis rate of GC cells increased in a dose-dependent manner after treatment with different concentrations of PD, reaching the maximum rate at 600 μg/ml. Apoptosis mainly comprised early apoptosis and late apoptosis. Therefore, the killing effect of PD on GC cells is mainly achieved by inducing cell apoptosis.

### 3.9 PD inhibits the PI3K/AKT, TNF, and IL-17 signaling pathways in GC cells

**Figure 8:**
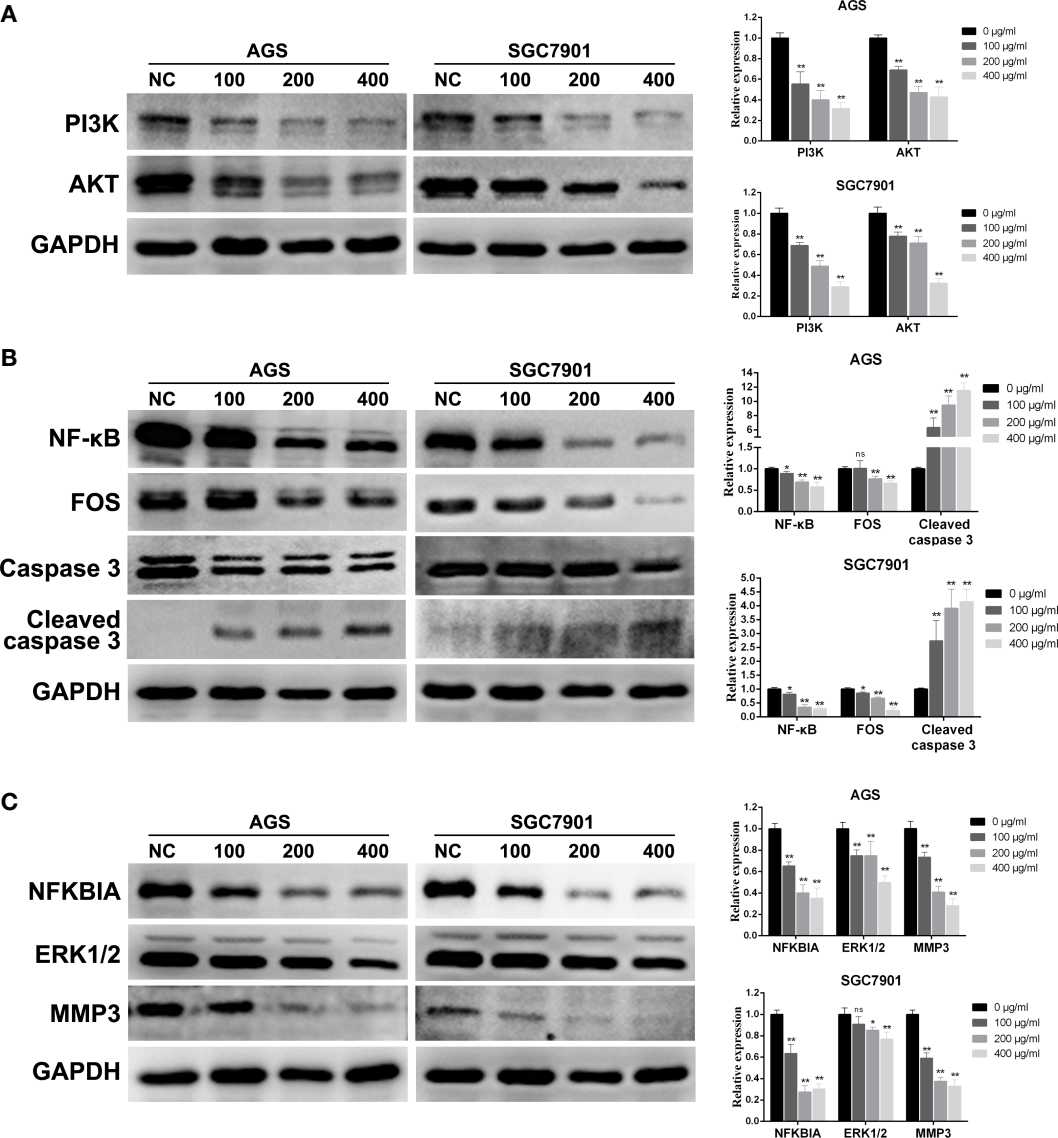
Changes in protein expression of GC cells treated with PD at different concentrations. **(A)** PI3K/AKT signaling pathway. **(B)** TNF signaling pathway. **(C)** IL-17 signaling pathway. *p < 0.05, **p < 0.01 versus the untreated group, ns, no significance.

To further explore the molecular mechanism of PD on GC, combined with the above network pharmacological analysis, we analyzed the key pathways involved in PD treating GC *via* Western blotting: the PI3K–AKT, TNF, and IL-17 pathways. As shown in **Figure 8A**, the levels of the key proteins PI3K and AKT in the PI3K/AKT pathway were significantly downregulated. The protein levels of NF-κB and FOS in the TNF pathway were also significantly downregulated, whereas those of caspase 3 splices were significantly upregulated (**Figure 8B**). In addition, the protein levels of NFKBIA, ERK1/2, and MMP3 in the IL-17 pathway were significantly downregulated (**Figure 8C**). Therefore, the killing effect of PD on GC cells and the inhibition of proliferation are mainly achieved *via the* PI3K–AKT, TNF, and IL-17 pathways, thereby achieving an antitumor effect.

## 4 Discussion

GC is a common digestive tract malignant cancer. The risk factors for GC contain *Helicobacter pylori* infection, age, high salt intake, and diets low in fruit and vegetables (1, 5). According to a report, the incidence and mortality of GC have been decreased in recent years; however, both incidence and mortality among young adults (aged < 50 years) are increasing (19). Moreover, GC patients are commonly associated with poor prognosis because of lacking specific symptoms in the early stage and most GC patients have been diagnosed in advanced stages. Nowadays, an increasing number of studies indicated that TCMs possess unique advantages in cancer treatment with improved drug efficiency and fewer side effects.

Previous modern pharmacological studies have reported that PD is characterized with better pharmacological and therapeutic effect against several cancers. PD acts on colon cancer *via* suppressing proliferation and metastasis, modulating differentiation and senescence, and promoting apoptosis (20). Moreover, PD showed synergy with 5-Fluorouracil to mediate colorectal cancer immunogenic cell death (21). However, the detailed mechanisms of PD anti-GC remain to be elucidated. Hence, network pharmacology and data analysis were performed to discover compounds, hub therapeutic target genes, and the potential mechanisms of PD anti-GC. In addition, experiments *in vitro* were utilized to further verify the potential mechanisms of PD against GC.

We screened a total of 34 PD compounds and 162 PD anti-GC target genes from multiple public databases. Several studies have reported that *Pulsatilla chinensis* saponins and its extracts, a major bioactive ingredient in PD, exert therapeutic effects by inducing cancer cell apoptosis, inhibiting tumor angiogenesis, and exhibiting anti-inflammatory properties (22–24). For example, Pulsatilla saponin A, an active ingredient in *P. chinensis*, can inhibit proliferation, induce DNA damage, and cause G2 arrest and the apoptosis of human hepatocellular carcinoma SMCC-7721 cells and pancreatic BXPC3 and SW1990 cancer cells (25). Guan et al. reported that three extracts of *P. chinensis* saponins, namely, Pulsatilla saponin A, raddeanoside R13, and Pulsatilla saponin D, can significantly inhibit the proliferation of NCI-H460 (human lung carcinoma), SMMC-7721 (human liver carcinoma), HCT-116 (human colorectal carcinoma), and U251 (human glioma) cells (26). Interestingly, in the present study, C–D–T network analysis revealed that quercetin, a common ingredient in *Cortex Phellodendri* and *Radix Pulsatillae*, had the highest degree among all the compounds and that it is a crucial bioactive compound in PD for treating GC. Quercetin, widely distributed in food, plants, and beverages, is a polyhydroxy flavonoid well known for its antioxidant, anti-inflammatory, antiviral, antimicrobial, and anticancer properties (27–29). Increasing evidence suggests that quercetin exhibits antitumor effects by inhibiting the cell cycle, cell proliferation, angiogenesis, and metastasis and promoting apoptosis (30–32). Ward et al. reported that quercetin exhibits anti-prostate cancer effects by suppressing cell survival transition and regulating related apoptotic pathways, including the ROS, AKT, and NF-κB pathways (33). Furthermore, Wang et al. reported that quercetin induces colon cancer cell apoptosis by increasing the gene expression of the apoptotic protein caspase 3 and decreasing that of the antiapoptotic protein Bcl-2 in a rat model of colon cancer (34). Moreover, quercetin suppressed cell migration and invasion *via the* GSK3β–β-catenin–ZEB1 signaling pathway (35).

The PPI network was constructed to investigate the hub target genes of PD anti-GC; the results revealed that JUN, ESR1, and AKT1, with higher degree values, might play important roles in the GC treatment process. Proto-oncogene JUN (c-Jun), an inducible transcription factor and mediator of oncogenic transformation, exerts various biological effects on apoptosis, proliferation, invasion, and migration (36–38). Lee et al. revealed that the overexpression of laminin subunit beta 1 facilitated the proliferation, invasion, and migration of GC cells *via the* ERK–c-Jun signaling pathway (39). Jia et al. reported that RPRD1B upregulation promotes fatty acid metabolism and lymph node metastasis in GC after activating the c-Jun–c-Fos–SREBP1 axis (40). ESR1, mainly encoding the estrogen receptor, is closely associated with breast and endometrial cancers; many studies have reported that the estrogen receptor plays a crucial anti-GC role by arresting the cell cycle and inducing apoptosis, invasion, and migration (41–43). AKT1, belonging to the AKT family, exerts various biological functions, including cell survival, proliferation, metabolism, and growth (44–46). Increasing evidence suggests that AKT is tightly associated with several cancer types *via* various signaling pathways, particularly the PI3K–AKT signaling pathway. Previous studies identified that the PI3K–AKT signaling pathway, one of the most dysfunctional signal transduction pathways in multiple cancer types, including GC, is involved in the cell cycle, cell proliferation, cell growth, cell differentiation, cellular metabolism, cell migration, angiogenesis, and apoptosis (45, 47–49). Similarly, in the present study, KEGG pathway enrichment analysis revealed that the PI3K–AKT signaling pathway, with a higher gene count enrichment, plays a critical role in the anti-GC effects of PD. Rong et al. reported that salidroside inhibits the proliferation and induces the apoptosis and autophagy of AGS cells *in vitro* and *in vivo* by targeting the PI3K–AKT–mTOR pathway (50). Moreover, the lncRNA AK023391 mediates the PI3K–AKT pathway to modulate tumorigenesis and invasion of MGC-803 and BGC-823 cells (51).

We performed molecular docking of the three target proteins, namely, JUN, ESR1, and AKT1, with higher degree values, and two compounds, namely, quercetin and isorhamnetin, to further validate the prediction results of network pharmacology. Docking experiments revealed that quercetin and isorhamnetin could stably bind with JUN, ESR1, and AKT1. Interestingly, quercetin and JUN exhibited the lowest binding energy, consistent with the results of PPI and C–D–T network analyses. Moreover, based on the results of KEGG pathway analysis, the PI3K–AKT, IL-17, and TNF signaling pathways were identified as the important cancer-related pathways of PD anti-GC. Therefore, *in vitro* experiments were performed in AGS and SGC7901 cells to further validate the biological functions of the three signaling pathways mentioned above when PD acts on GC. Previous studies have reported that the PI3K–AKT, IL-17, and TNF signaling pathways exert anticancer properties by inhibiting proliferation, arresting the cell cycle, and promoting apoptosis. The cellular experiments in the present study also verified that PD exerts similar anti-GC effects on AGS and SGC7901 cells. This finding is consistent with the prediction results of network pharmacology.

The present study has several limitations that should be highlighted. First, these online public databases are continuously updated; therefore, other updated bioactive components and target genes may have not been included in this study. Moreover, the content of the 34 PD compounds remains unclear; therefore, quantitative analysis should be performed and compounds with anti-GC effects should be identified in future studies. Furthermore, the absorption pathways, structural domains, and metabolism form of PD against GC should be elaborated. Finally, the anti-GC effects of PD should be further validated in other GC cells and animal models and *via* clinical trials.

## 5 Conclusion

In summary, this is the first report of PD in the treatment of GC based on network pharmacology, and experiments *in vitro* were performed to validate the prediction results of network pharmacology. Our work demonstrated that PD inhibited GC cell proliferation and induced apoptosis *via the* PI3K–AKT signaling pathway, IL-17 signaling pathway, and TNF signaling pathway, which provide valuable evidence of the role of PD in cancer development. Additionally, the present study provided a comprehensive and promising approach to explore bioactive compounds, hub genes, and the potential mechanisms of TCM in the treatment of diseases.

## Data availability statement

The original contributions presented in the study are included in the article/**Supplementary Material**. Further inquiries can be directed to the corresponding author.

## Ethics statement

This study was approved by the Medical Ethics Committee of Guangdong Second Provincial General Hospital. All experiments were conducted following the study protocol.

## Author contributions

All authors contributed to the study conception and design. Material preparation, data collection, and analysis were performed by FK, MQ, SY and XC. The first draft of the manuscript was written by SH and all authors commented on previous versions of the manuscript. All authors contributed to the article and approved the submitted version.

## Supplementary material

Supplementary Figure 1 The PI3K-AKT signaling pathway. The PI3K-AKT signaling pathway may play an important role in the treatment of GC. Nodes in red stand for PD anti-GC related genes.

Supplementary Figure 2 The TNF signaling pathway. The TNF signaling pathway may play an important role in the treatment of GC. Nodes in red stand for PD anti-GC related genes.

Supplementary Figure 3 The IL-17 signaling pathway. The IL-17 signaling pathway may play an important role in the treatment of GC. Nodes in red stand for PD anti-GC related genes.

Supplementary Table 1 GO analyze of PD anti-GC.

Supplementary Table 2 KEGG analyze of PD anti-GC.
