# Supplementary figures and images for: Determining the mechanism of pulsatilla decoction for treating gastric cancer: a network pharmacology-based study
Source: Front Oncol. 2023 Jun 9;13:1174848. doi: 10.3389/fonc.2023.1174848 (PMC10289198; doi:10.3389/fonc.2023.1174848)

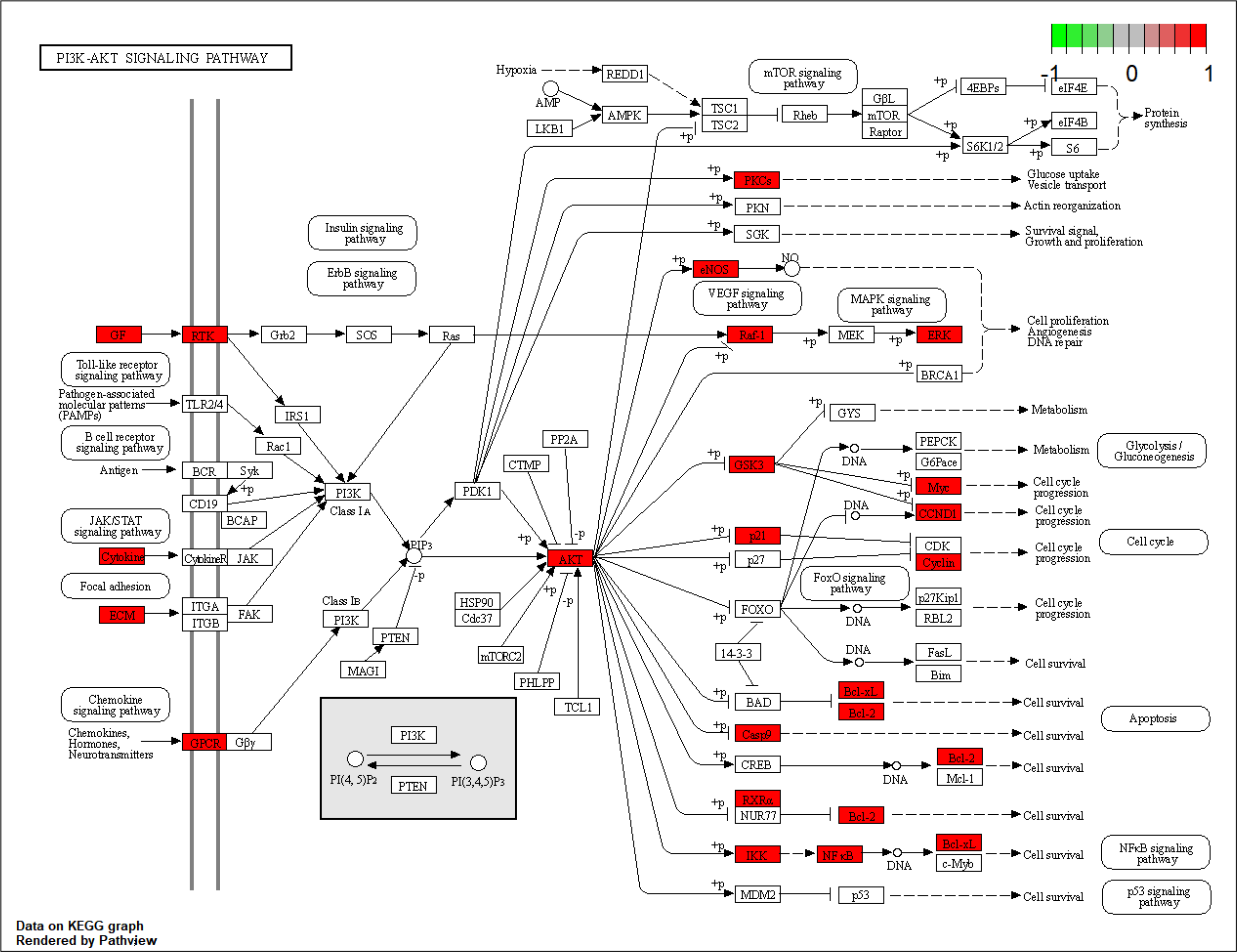

Supplement: Supplementary Figure 1 — The PI3K-AKT signaling pathway. The PI3K-AKT signaling pathway may play an important role in the treatment of GC. Nodes in red stand for PD anti-GC related genes. [file Image_1.tif]

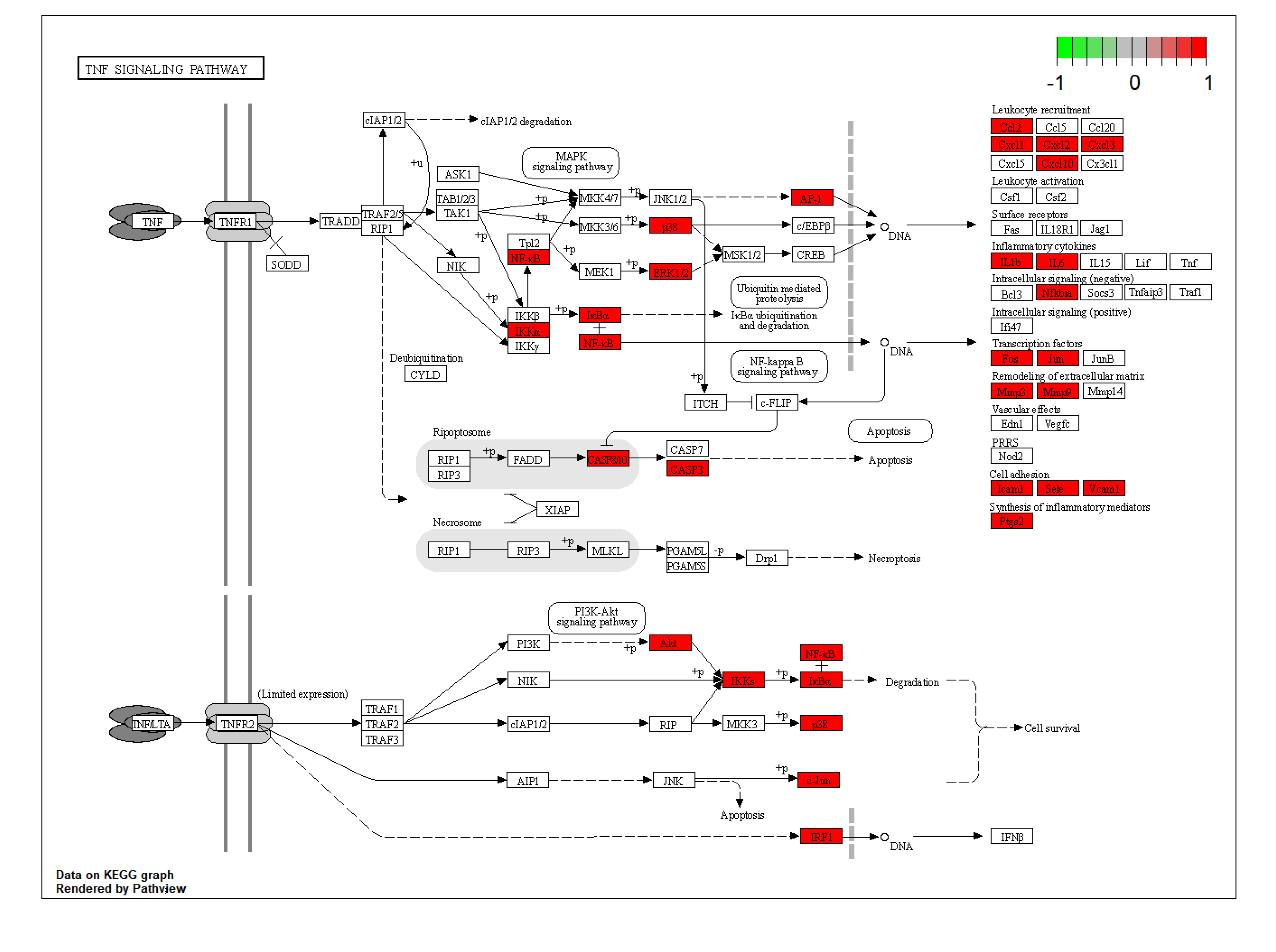

Supplement: Supplementary Figure 2 — The TNF signaling pathway. The TNF signaling pathway may play an important role in the treatment of GC. Nodes in red stand for PD anti-GC related genes. [file Image_2.tif]

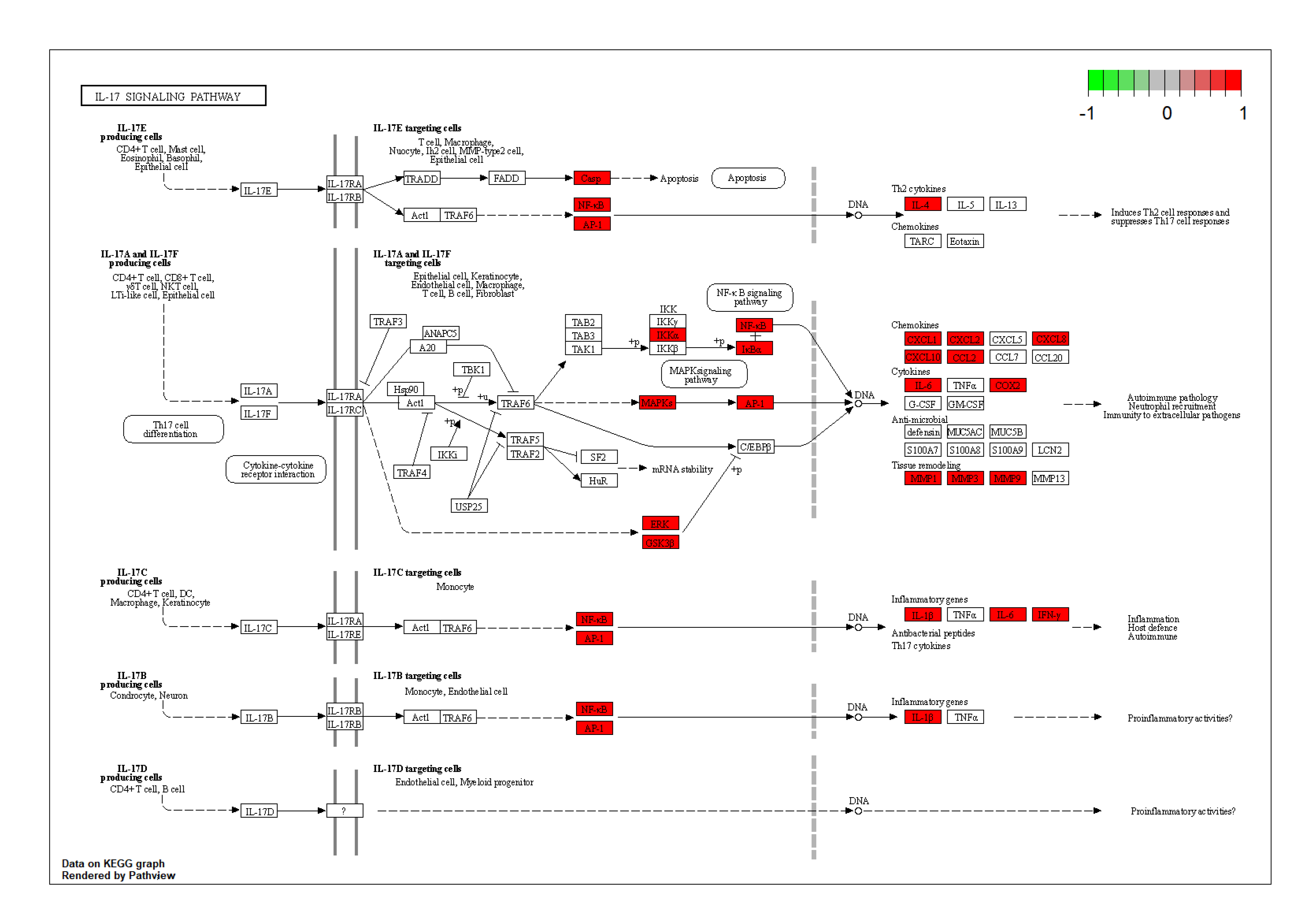

Supplement: Supplementary Figure 3 — The IL-17 signaling pathway. The IL-17 signaling pathway may play an important role in the treatment of GC. Nodes in red stand for PD anti-GC related genes. [file Image_3.tif]
